# Breathless Nights and Cardiac Frights—How Snoring Is Breaking Hearts

**DOI:** 10.3390/biomedicines12122695

**Published:** 2024-11-26

**Authors:** Michael Wester, Simon Lebek

**Affiliations:** Department of Internal Medicine II, University Hospital Regensburg, 93053 Regensburg, Germany; michael.wester@ukr.de

## 1. Sleep-Disordered Breathing Is Highly Relevant in the Cardiovascular Field

While your nightly symphony may be testing your loved one’s patience, it could also be giving your own heart reasons to complain. In fact, more than one billion people worldwide have sleep-disordered breathing (SDB), which affects not only their partner’s sleep but also their own health [1,2]. Thus, this Special Issue, “Sleep-Disordered Breathing and Cardiovascular Diseases”, aims to address this clinical area and contribute novel insights.

Among other issues, SDB is associated with hypertension [3] and cardiac arrhythmias, such as atrial fibrillation [4,5], that may lead to subsequent strokes [6]. SDB is also a highly prevalent disorder in patients with heart failure; it is seen in approximately 50% of these patients [7,8]. Central sleep apnea is very common in heart failure with reduced ejection fraction (HFrEF) [7]. For heart failure with preserved ejection fraction (HFpEF), it is more complicated due to various HFpEF phenotypes. Notably, the “metabolic, obese phenotype” has a high clinical overlap with patients with obstructive sleep apnea, which is reviewed in an article in this Special Issue [9].

## 2. Current Therapeutic Strategies and Patient Prognosis

Poor sleep and SDB are associated with an increased risk of cardiovascular morbidity [4,5,6,10]. Loud snoring can be a sign of silent killers such as strokes, myocardial infarctions, and heart failure. Apart from marital discomfort, patients may suffer from daytime sleepiness. If this is due to obstructive sleep apnea (OSAS), it can be effectively treated with lifestyle interventions, mandibular advancement splints, oropharyngeal surgery, or continuous positive airway pressure therapy (CPAP) [11]. Unfortunately, patients’ compliance with recommended lifestyle interventions (e.g., diet or exercise) is typically low [12,13,14].

The stress that is imposed on the heart by OSAS causes and worsens cardiac disease. For example, increased myocardial remodeling with an impaired cardiac structure and diastolic dysfunction was reported in SDB patients after a myocardial infarction [15,16]. However, SDB is a treatable risk factor. The recent TEAM-ASV-I trial showed that CPAP treatment in SDB patients after an acute myocardial infarction reduced the infarct size and improved the myocardial salvage index [17]. This proves that the detection and consequent treatment of SDB are crucial especially in patients with high cardiovascular risk and acute cardiac stress. However, many questions remain unanswered, for example, how SDB patients with reduced ejection fraction should be treated in the light of the SERVE-HF trial, where all-cause and cardiovascular mortality was increased in patients subjected to adaptive servo-ventilation [18]. Moreover, it is unclear how SDB treatment influences the development of heart failure with preserved ejection fraction [9].

As obesity is a causal risk factor for obstructive sleep apnea, new developments in pharmacological weight loss, for example, with GLP1-agonists, will likely also reduce the burden of SDB in these patients. Early clinical studies show promising results [19]. There are many small studies exploring different pharmacological treatment options for SDB; however, none of these have yet entered broad clinical practice [20,21]. Thus, current therapeutic strategies are mainly limited to CPAP. However, this approach recently failed to reduce the burden of atrial fibrillation [22] or the long-term incidence of adverse cardiovascular events [23]. This may be due to the limited effectiveness of the treatment in these cohorts but also due to patients’ poor adherence to CPAP, which is typical in patients with cardiovascular diseases [24]. This highlights the need for the development of new therapeutic strategies for SDB patients, which requires detailed insights into the underlying pathomechanisms in order to identify potential molecular targets.

## 3. Pathomechanisms

Since SDB is a systemic disorder that causes intermittent hypoxia, increased β-adrenergic stimulation, and intrathoracic pressure swings, it leads to a multitude of dysregulated processes [2,5,9]. These range from oxidative stress and inflammation to metabolic alterations and structural remodeling, such as fibrosis [2,5,9,25]. Another central mechanism is a dysregulation of the cellular Na^+^ and Ca^2+^ homeostasis that is mediated by the cardiac stress-responsive enzyme Ca^2+^/calmodulin-dependent protein kinase IIδ (CaMKIIδ) [26,27,28,29]. In patients and mice with SDB, we found an increased myocardial production of reactive oxygen species (ROS), leading to a pathogenic overactivation of CaMKIIδ via the oxidation of two critical methionines [26,27,28,29]. This resulted in an increased sarcoplasmic reticulum Ca^2+^ leak, decreased Ca^2+^ transients, and an enhanced late Na^+^ current that culminated in cardiac arrhythmias and contractile dysfunctional [26,27,28,29]. This Special Issue offers novel insights into the pathomechanisms of SDB. Pec et al. found that in patients after acute myocardial infarction, the presence of obstructive SDB is independently associated with increased inflammation (high-sensitivity C-reactive protein) and fibrosis (procollagen III amino-terminal propeptide) [30]. Moreover, Chetan et al. demonstrated that the endothelial biomarker VCAM-1 is associated with high cardiovascular risk in patients with obstructive sleep apnea [31]. The authors further concluded that conventional risk stratification scores could be improved by incorporating biomarkers such as VCAM-1 [31]. Even though both studies revealed interesting insights into the mechanisms of SDB and cardiovascular disease in patients, there is still much more to learn.

## 4. Current Developments

As the co-existence of SDB and cardiovascular disease is high and the number of sleep laboratories is limited, reliable and simple testing for SDB remains an unmet need. Recent developments in the field of wearables and smartwatches might offer a solution. These devices can already detect atrial fibrillation, and their use is recommended in recent guidelines [32,33]. The next step will be registering sleep and SDB [34].

The advantages are obvious, as unobtrusive data collection every night allows for a detailed assessment of sleep and individual variations in sleeping patterns. Sleep, sleep stages, and apneas cannot be directly measured by sensors and are therefore predicted by complex algorithms incorporating a multitude of signals (such as heart rate variability, movement, and oxygen saturation). However, these algorithms are AI-based black boxes, and corporate secrecy prevents academic research. The use of wearable technology to screen patients for SDB is very promising, as it represents a low-threshold method that evaluates sleep in the comfort of an individual’s own home, provides long-term data, and may even provide new parameters. However, the external validity of these data and the inter-vendor differences have yet to be thoroughly investigated and addressed [35,36,37].

## 5. Conclusions

SDB is widely underestimated, under-diagnosed, and insufficiently understood. It affects patients on a physiological, prognostic, and very personal level. The field of sleep medicine has seen huge progress in the production of a steadily growing body of evidence regarding pathomechanisms and diagnostic and treatment options for SDB patients. This is highlighted in this Special Issue of *Biomedicines*, “Sleep-disordered Breathing and Cardiovascular Diseases”. The intricate interplay between sleep and cardiovascular disease is manifold and highly relevant but potentially treatable. It affects not only the patient’s cardiac health but also their direct environment, as well as their partner. Thus, by silencing snores, we may be able to mend two hearts at once—the one that beats and the one that loves.

## Data Availability

Not applicable.
